# Identification of Dermatological Conditions Manageable in Primary Care: A Multidisciplinary Consensus Analysis

**DOI:** 10.3390/healthcare14111596

**Published:** 2026-06-05

**Authors:** Bekir Aktura, Esmehan Ayşit, Feyza Bayram Çatak, Ece Uysal Kasap, Pınar Kıran, Beyza Arpacı Saylar, Zafer Türkoğlu, Defne Özkoca, Nazlı Caf, Mehmet Onur Gökalp, Hediye Eker, Mehmet Burhan Küçükoğlu

**Affiliations:** 1 Department of Family Medicine, Faculty of Medicine, Atlas University, Istanbul 34408, Türkiye; 2 Presidency of Public Health Services, Istanbul Provincial Health Directorate, Istanbul 34142, Türkiye; 3 Arnavutköy Darülaceze Vocational School of Health Services, University of Health Sciences, Istanbul 34277, Türkiye; 4 Eyüpsultan District Health Directorate, Istanbul 34050, Türkiye; 5 Department of Dermatology, Başakşehir Çam and Sakura City Hospital, Istanbul 34480, Türkiye

**Keywords:** primary care, dermatology referral, inter-rater agreement, family medicine, teledermatology, digital health

## Abstract

**Highlights:**

- Across a multidisciplinary commission assessment, 51.5% of tertiary dermatology outpatient visits were judged manageable in primary care—27.5% by a family physician directly and a further 24.0% with teledermatology support.
- Specialist self-referral was very common: 82.2% of patients reached the tertiary dermatology clinic without any prior family-physician visit, consistent with a health system that does not require primary-care gatekeeping.
- Strengthening dermatology training and diagnostic confidence in family medicine is the primary lever for managing common skin conditions earlier; teledermatology is best understood as one structured decision-support pathway that complements, rather than replaces, this capacity.
- Inter-commission agreement was moderate overall (κ = 0.430) and lower in pediatric cases (κ = 0.312), indicating where additional support and calibration are most needed.

**Abstract:**

**Background**: Dermatological diseases account for a substantial share of primary-care visits, yet many are referred to secondary or tertiary services. A notable proportion of dermatology outpatient visits may instead be manageable within primary care. We evaluated whether patients assessed at the tertiary level could have been managed in primary care, in order to inform referral optimization. **Methods**: In this retrospective, cross-sectional study, the records of 400 patients who presented to the dermatology outpatient clinic of a city hospital in Istanbul between May, and July 2025 were analyzed. Each case was independently classified by three multidisciplinary commissions; each commission comprised a dermatologist, a family medicine specialist, and a general practitioner. Each commission produced a single panel-level management decision per case, and inter-commission agreement across the three commissions was assessed with Fleiss’ kappa. **Results**: Of the 400 patients, 58.8% were female and the mean age was 31.0 ± 17.6 years; 27.5% (*n* = 110) were pediatric (aged 0–18 years). Overall, 51.5% of cases were judged manageable in primary care (27.5% by a family physician directly and 24.0% with teledermatology support); this was 55.5% in pediatric and 50.0% in adult patients. Only 17.8% of patients had consulted a family physician in the 7 days before attendance. The most frequent diagnoses were acne, dermatitis, and superficial fungal infections. Inter-commission agreement was moderate overall (κ = 0.430; *p* < 0.001) and lower in pediatric (κ = 0.312) than adult (κ = 0.471) cases. **Conclusions**: A substantial proportion of tertiary dermatology visits could plausibly be managed in primary care. Realizing this potential depends primarily on strengthening primary-care dermatology capacity, with teledermatology serving as one structured, supportive pathway.

## 1. Introduction

Skin diseases constitute a significant portion of primary-care visits and are a common reason for referral to secondary and tertiary care worldwide. Family physicians are frequently responsible for the initial diagnosis, treatment, and referral of patients with dermatological complaints [1,2]. Effective management of common skin conditions in primary care can improve access, reduce avoidable referrals, and optimize the use of health-care resources [2,3].

Despite the high prevalence of dermatological presentations in primary care, diagnostic, treatment, and referral patterns differ substantially among non-dermatologist physicians [1,3,4]. Limited dermatology exposure during undergraduate and postgraduate training, together with less day-to-day clinical experience, contributes to diagnostic uncertainty [3,5]. As a consequence, patients are sometimes referred to tertiary centers for conditions that could be managed at the primary-care level [2,3], even though a considerable proportion of specialist dermatology referrals are considered avoidable [2,3,4]. Such referrals can raise costs, lengthen waiting times, and reduce timely access for patients with more serious disease [4].

These dynamics are shaped by how each health system organizes first-contact care. In Türkiye, family medicine was established as the backbone of primary care through the Health Transformation Program from 2010 onward, and family physicians provide accessible, no-cost first-contact services. However, the system does not operate a formal gatekeeping model: patients may present directly to secondary and tertiary outpatient clinics, including dermatology, without a prior referral. This combination of strong primary-care infrastructure and unrestricted direct specialist access provides an informative setting in which to examine how many tertiary dermatology visits could, in principle, have been handled earlier in primary care, and how often patients bypass that level entirely.

Comparative studies that evaluate management-level agreement for the same patient population across dermatologists and primary-care physicians remain limited [1,3]. Most prior work has focused on pairwise diagnostic concordance or referral outcomes rather than on multidisciplinary, management-oriented decisions that mirror routine governance. Addressing this gap is essential to improve referral pathways and to define more precisely the role of primary care and of digital tools such as teledermatology [2,6].

Therefore, the aim of this study was to retrospectively analyze the dermatology consultations of 400 patients evaluated at a tertiary center, to determine—through independent, multidisciplinary commission assessment—whether these patients could have been managed within primary care, and to characterize the conditions and patient groups for which primary-care management (with or without teledermatology support) is most and least likely to be agreed upon.

## 2. Materials and Methods

### 2.1. Study Design

This was a retrospective, cross-sectional study. The records of patients who attended the dermatology outpatient clinic of a city hospital in Istanbul during May, June, and July 2025 were analyzed retrospectively.

### 2.2. Data Sources and Data Collection

Data were obtained from the hospital record system and the Istanbul Provincial Health Directorate records. The variables examined included age, sex, presenting complaint, medical history, physical-examination findings, clinical diagnosis, ICD-10 diagnosis codes, physician notes, and decision and treatment information.

For each patient in the sample, system records were checked to determine whether the patient had visited a primary-care (family medicine) facility within the 7 days preceding the dermatology visit. A 7-day window was chosen a priori as a clinically meaningful interval over which a recent primary-care contact would plausibly relate to the same episode of skin complaint, while remaining short enough to avoid capturing unrelated visits. Because this threshold is necessarily somewhat arbitrary, we treat it as a definitional choice and discuss its implications under Limitations; the prior-visit rate is therefore a conservative lower bound for any longer look-back period.

The required sample size was based on teledermatology studies reporting that approximately 53% of dermatology consultations could be managed at the primary-care level [7]. Over the three-month period, there were 66,416 total dermatology outpatient examinations; after applying the exclusion criteria below, 6970 records were excluded, leaving 59,446 eligible outpatient examinations. With a 95% confidence level and a 5% margin of error applied to this eligible population, the minimum sample size was *n* = 381; allowing for possible data loss, the final target was *n* = 400. Cases were drawn from the 59,446 eligible examinations by systematic sampling (sampling interval ≈ 1 in 149).

Exclusion criteria were: patients seen in unrelated outpatient clinics; patients referred to consultation or health-board clinics; patients who registered but were not examined; hospitalized inpatients; and foreign nationals (whose primary-care records were not reliably linkable).

### 2.3. Commission-Based Evaluation Process and Rater Roles

The 400 cases were assessed by three independent commissions. Each commission comprised three physicians: a dermatology specialist, a family medicine specialist (i.e., a physician with completed specialty training in family medicine, working as a family physician), and a general practitioner (a physician without specialty training in family medicine). This composition was deliberately mixed so that each panel-level decision would reflect realistic, multidisciplinary primary-care-to-specialist deliberation rather than the judgment of a single discipline; it mirrors the range of clinician experience that exists in the Turkish primary-care workforce, in which both family medicine specialists and general practitioners deliver first-contact care.

Commission members reviewed each patient record independently and classified the case into one of three categories:

1. Can be monitored/treated by a family physician.
2. Can be monitored/treated by a family physician with teledermatology consultation.
3. Must be referred to a dermatologist.

“Manageable in primary care” was defined as a condition that could be diagnosed, treated, and followed longitudinally by a family physician using standard treatment protocols, without requiring in-person specialist evaluation at the time of presentation (categories 1 and 2 combined).

To improve consistency before formal rating, evaluators were briefed with a common written instruction set defining the three categories and the meaning of “manageable in primary care,” and a small set of pilot cases was reviewed jointly to calibrate interpretation; these pilot cases were not part of the 400 analyzed records. Each commission then rated cases independently and produced a **single panel-level decision per case** using the following rule: if any member assigned “3,” the commission decision was “3”; otherwise, if any member assigned “2,” the decision was “2”; if all members assigned “1,” the decision was “1.” This conservative (referral-prioritizing) rule was adopted intentionally to prioritize patient safety and minimize under-referral, reflecting risk-averse clinical governance. We acknowledge that it is asymmetric: it can only move a case toward referral, and therefore tends to underestimate, rather than overestimate, the proportion judged manageable in primary care. The 51.5% reported here is thus a conservative lower bound.

### 2.4. Ethical Approval

Local ethics committee approval and institutional permissions were obtained (KAEK/10.12.2025.395). All data were anonymized, and no personally identifiable information was included in the analysis. Data were used solely for scientific purposes and stored in accordance with privacy principles. The study was conducted in accordance with the Declaration of Helsinki.

### 2.5. Data Analysis

Descriptive statistics (percentages, means, standard deviations, minima, and maxima) were used. IBM SPSS 27.00 was used for statistical analysis. Patients were classified as pediatric if aged 0–18 years inclusive and as adult if older than 18 years. The unit of analysis for the reliability assessment was the commission (panel): each of the three commissions contributed one decision per case, and inter-commission agreement across these three panel-level decisions was assessed with Fleiss’ kappa (three raters, 400 subjects, three categories). Kappa was interpreted as: <0, poor; 0.01–0.20, slight; 0.21–0.40, fair; 0.41–0.60, moderate; 0.61–0.80, substantial; 0.81–1.00, almost perfect. Statistical significance was set at *p* < 0.05.

## 3. Results

**Figure 1:**
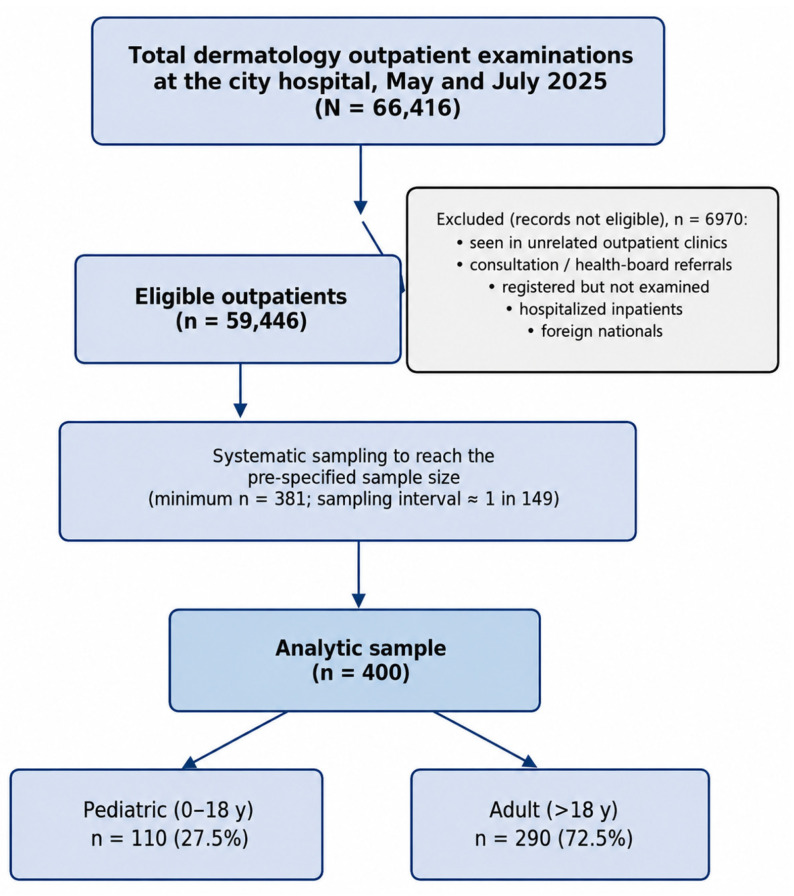
Participant flow. Of 66,416 total dermatology outpatient examinations over the three-month period, 6970 were excluded per the criteria, leaving 59,446 eligible examinations; *n* = 400 cases were drawn by systematic sampling (sampling interval ≈ 1 in 149; 110 pediatric, 290 adult). Per-category exclusion counts were not separately retrievable, so the aggregate excluded total is shown.

Among the 400 patients, 58.8% were female and the mean age was 31.0 ± 17.6 years (range 0–82). Pediatric patients (aged 0–18 years) accounted for 27.5% (*n* = 110) and adults for 72.5% (*n* = 290). The derivation of the analytic sample is shown in Figure 1.

**Figure 2:**
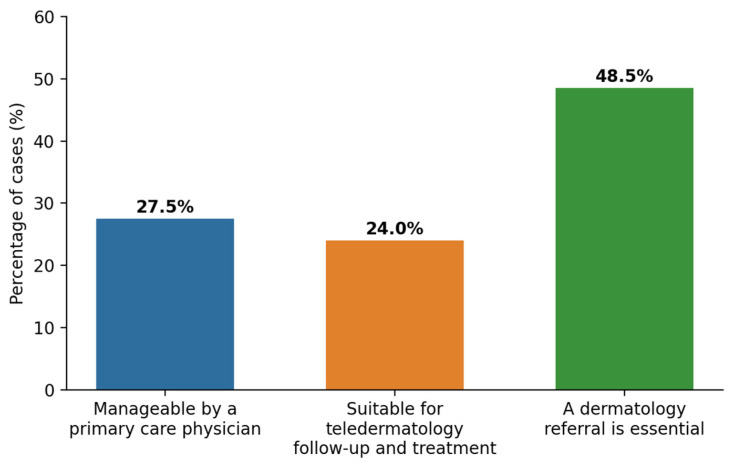
Distribution of dermatology presentations by management category according to commission decisions (values shown to one decimal place).

Across the three commissions, 51.5% of cases were judged manageable in primary care: 27.5% by a family physician directly and 24.0% with teledermatology support, while 48.5% were judged to require dermatology referral (Figure 2).

By age group, among pediatric cases, 22.7% were judged manageable directly by a family physician and 32.7% with teledermatology, whereas among adults, the corresponding figures were 29.3% and 20.7%. Overall, 55.5% of pediatric and 50.0% of adult patients were judged manageable in primary care.

**Figure 3:**
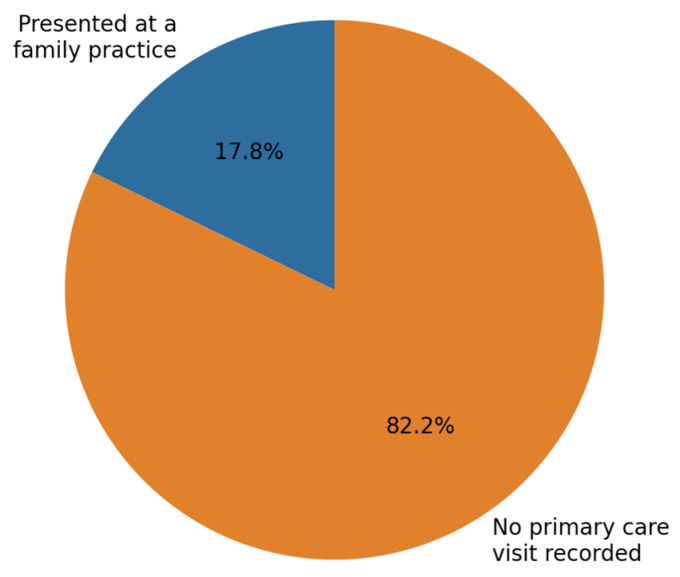
Family-physician visits in the 7 days prior to the dermatology outpatient visit (values shown to one decimal place).

Regardingprior primary-care contact, only 17.8% (*n* = 71) of patients had visited their family physician in the preceding week, whereas 82.2% (*n* = 329) attended the dermatology clinic directly without a prior primary-care visit (Figure 3).

In the subgroup analysis, prior-week family-physician contact was recorded for 11.8% (*n* = 13) of patients deemed manageable by a family physician, 19.8% (*n* = 19) of those deemed suitable for teledermatology-supported management, and 20.1% (*n* = 39) of those requiring dermatology referral; the majority in all three groups had no prior-week primary-care visit (Table 1).

The most frequent presenting diagnoses were acne, dermatitis (including seborrheic dermatitis), and superficial fungal infections (dermatophytosis/tinea/pityriasis versicolor). A consolidated breakdown of all presentations by diagnostic group, together with the management-category distribution for each group, is provided in Table 2; the complete, finer-grained breakdown is given in Supplementary Table S1.

Inter-commission agreement was moderate overall, with a Fleiss’ kappa of 0.430 (*p* < 0.001). In the subgroup analysis, agreement was lower in pediatric patients (κ = 0.312, fair) and higher in adult patients (κ = 0.471, moderate) (both *p* < 0.001). Consistent with this, the proportion of cases in which the three commissions did not fully agree was higher in pediatric (60.0%) than in adult (46.9%) cases (overall 50.5%).

Disagreement was concentrated in a limited set of diagnoses. Among groups with at least 10 cases, the highest rates of inter-commission disagreement were observed for acne (≈61%), scabies (55%), viral warts (53%), and pruritus (46%)—conditions in which the boundary between primary-care management and specialist referral is genuinely judgment-dependent (e.g., acne severity grading and treatment escalation, or scabies in the presence of secondary infection). In pediatric cases specifically, acne and viral warts accounted for a disproportionate share of disagreements, in line with the lower overall pediatric kappa.

## 4. Discussion

Our results indicate that a substantial proportion of tertiary dermatology outpatient visits could plausibly be managed within primary care, either directly by a family physician or with teledermatology-supported consultation. By showing that more than half of presentations fall within a multidisciplinary panel’s definition of primary-care-manageable scope, our findings support efforts to strengthen first-contact care and optimize specialist referral pathways in systems facing rising demand. This proportion should be read as a conservative estimate, given the referral-prioritizing decision rule described in the Methods.

The estimate aligns with studies indicating that roughly 45–65% of dermatology referrals do not require in-person specialist evaluation when appropriate triage is in place [8,9]. Contemporary teledermatology research increasingly frames the modality not as a straightforward substitute for face-to-face care but as a clinical-governance tool supporting decision-making, risk stratification, and care coordination [8,10]. Our findings add to this literature by suggesting that teledermatology-supported primary-care management is feasible across both adult and pediatric populations, albeit with differing levels of agreement.

Beyond estimating the manageable proportion, this study introduces a commission-based, multidisciplinary evaluation model that reflects real-world decision-making. Unlike prior work centered on pairwise diagnostic concordance or referral outcomes, our approach integrates management-level decisions across professional roles, capturing the complexity of referral appropriateness in routine practice and providing a pragmatic framework for identifying conditions suitable for primary care [11].

The predominance of acne, dermatitis, superficial fungal infections, and follicular disorders mirrors global ambulatory-dermatology patterns [12,13]. These are generally high-frequency, lower-complexity diagnoses that respond to standardized algorithms and longitudinal follow-up—strengths of primary care [13]. Yet, as our diagnostic breakdown shows, even within these groups, a meaningful minority were judged to require referral (for example, only ~54% of acne and ~9% of viral-wart presentations were deemed primary-care-manageable), underscoring that disease label alone does not determine management and that severity and morphology matter.

Limited undergraduate and postgraduate dermatology exposure among primary-care physicians is a recognized contributor to diagnostic uncertainty and referral behavior [14,15]. In this context, teledermatology may help family physicians validate clinical impressions, refine management, and safely defer unnecessary referrals [10,16]. It has also been hypothesized that teledermatology-supported care could enhance learning through case-based feedback and improve diagnostic accuracy over time [14]; however, our cross-sectional design cannot test this, and we present it only as a hypothesis for prospective evaluation rather than as a demonstrated effect of our data.

A notable finding is the low rate of primary-care consultation before tertiary attendance: more than four in five patients accessed specialist care without first seeing a family physician. Similar bypass patterns are documented in systems with weak gatekeeping or unrestricted specialist access, where self-referral is common [17,18]. In the Turkish setting, direct access to specialist outpatient clinics—and the availability of private-sector dermatology for patients who can pay out of pocket—likely contributes to this pattern, and our administrative records may not capture private-sector or out-of-network primary-care contacts. Such bypass strains specialist services and can undermine the continuity and coordination roles central to effective primary care.

From a systems perspective, avoidable specialist utilization is associated with longer waits, higher costs, and reduced access for complex or high-risk patients [17,18]. Prior work suggests that teledermatology-based triage can reduce unnecessary referrals and improve access in some settings, although the magnitude and consistency of benefit depend on workflow design, case selection, and the local access structure [7,10,19]. Our data identify a large pool of patients who might be safely managed outside tertiary care, providing empirical grounding for such efforts.

Recent teledermatology literature helps contextualize these findings, but the newer studies represent different evidence types and should be interpreted accordingly. Hamid et al. reported that a high proportion of teledermatology-assessed primary-care skin-lesion referrals could be managed without face-to-face review [20]. Jairath et al. prospectively validated a complexity-based triage score in 100 consecutive teledermatology cases and showed that low-complexity cases can be identified reproducibly, with primary-care management appearing feasible in that quality-filtered subset rather than across all referrals [21]. By contrast, McDowell et al. examined provider perspectives in a pediatric, store-and-forward, safety-net program rather than patient-level clinical outcomes [22], and Bouton et al. reported mixed results in a cluster-randomized primary-care trial of smartphone photograph transmission, with no significant benefit for the primary endpoint (lesions ultimately requiring resection) [19]. Taken together, these studies support teledermatology as a context-dependent governance and decision-support tool rather than a uniform substitute for in-person care.

Inter-commission agreement was moderate overall and higher for adults than for children. This is consistent with evidence that pediatric dermatology poses greater diagnostic and management challenges owing to age-specific presentations, communication constraints, and heightened medicolegal concern [23]. The lower pediatric agreement—and the concentration of disagreement in conditions such as acne and viral warts—highlights where structured support and evaluator calibration are most needed. Because evaluators made management-level rather than single-diagnosis judgments, moderate agreement is expected and reflects the inherent complexity of referral decisions; it should not be interpreted as diagnostic unreliability.

If teledermatology is to be deployed, our findings suggest it is most valuable for specific groups and circumstances rather than universally: for lower-complexity inflammatory and infectious conditions in which a family physician needs confirmation rather than transfer of care; for pediatric cases where diagnostic uncertainty is higher; and for patients who would otherwise bypass primary care. Equity considerations are important, as the benefits of digital pathways depend on reliable connectivity, image quality, and patient access to primary care; poorly implemented teledermatology could widen rather than narrow disparities. Realizing benefit therefore depends primarily on strengthening primary-care dermatology capacity, with teledermatology as a complementary, well-governed pathway.

### 4.1. Implications for Practice and Policy

First, the findings reinforce the need to strengthen dermatology competencies within family-medicine training and continuing professional development, particularly for common inflammatory and infectious conditions; structured continuing-education programs and case-based feedback are plausible levers. Second, they support integrating structured teledermatology pathways into primary-care workflows as governed components of referral pathways rather than optional add-ons. Third, they underscore the value of system-level measures that encourage primary care as the first point of contact, thereby reducing specialist bypass. Recent policy frameworks emphasize that digital tools are most effective when aligned with broader primary-care strengthening rather than implemented in isolation [24].

If implemented in this setting, teledermatology should operate as an asynchronous store-and-forward service embedded within routine working hours, with family physicians submitting standardized histories and clinical photographs through an existing digital platform and designated tertiary-center dermatologists providing structured responses within a defined turnaround time. Framed this way, teledermatology functions as a governed referral-support mechanism rather than an ad hoc substitute for face-to-face care. Such an approach may improve equity for patients who would otherwise bypass primary care, but only if image-quality standards, response times, and access to follow-up pathways are explicitly defined; without these safeguards, poorly implemented digital pathways could widen rather than narrow disparities.

### 4.2. Strengths and Limitations

Strengths include a sizeable sample, real-world tertiary-care data, and independent multidisciplinary assessment. Several limitations should be acknowledged. First, the design is retrospective and record-based: commissions judged manageability from documented information rather than live examination, which may introduce misclassification, and the absence of photographs or in-person assessment could bias decisions in either direction. Second, the conservative decision rule is asymmetric and can only shift cases toward referral, so the manageable proportion is a lower bound. Third, the 7-day look-back window for prior primary-care contact is a definitional choice; a longer window would likely increase the recorded prior-visit rate, and administrative data may miss private-sector or out-of-network visits, so the 17.8% figure is a conservative estimate of true prior contact. Fourth, systematic sampling from a single tertiary center in Istanbul limits generalizability, particularly to systems with different access or referral structures, and to private-sector settings; in addition, any periodicity in the registration sequence could in principle introduce selection bias, so the representativeness of the sample should be interpreted cautiously. Fifth, the design precludes evaluation of patient outcomes had primary-care management actually been implemented. Prospective, multicenter studies incorporating outcomes, cost analyses, image-based assessment, and patient-reported experience would strengthen the evidence base.

A further limitation concerns the panel composition. Our mixed-specialty design captures pooled, multidisciplinary judgment rather than specialty-specific referral thresholds. Although panel members first reviewed each case independently, combining dermatologist, family medicine specialist, and general practitioner perspectives within a single decision structure may have attenuated discipline-specific differences that would be apparent in specialty-specific panels; the agreement reported here should therefore be read as agreement between mixed commissions rather than between disciplines.

## 5. Conclusions

A large proportion of tertiary dermatology outpatient visits could plausibly be managed within primary care. Capturing this potential depends primarily on strengthening primary-care dermatology capacity—through training, continuing education, and clear referral criteria—with teledermatology serving as one structured, equitable, and well-governed pathway rather than a stand-alone solution. Addressing the high rate of specialist bypass is an additional priority for improving the efficiency and sustainability of dermatological care.

## Figures and Tables

**Table 1:** Prior-week family-medicine visits according to commission management decision.

Prior-Week FM Visit	Manageable by Family Physician	Manageable with Teledermatology	Referral to Dermatologist
Yes—*n* (%)	13 (11.8%)	19 (19.8%)	39 (20.1%)
No—*n* (%)	97 (88.2%)	77 (80.2%)	155 (79.9%)

**Table 2:** Presenting diagnoses by consolidated group, with commission management-category distribution and the proportion judged manageable in primary care (categories 1 + 2).

Diagnostic Group	*n*	%	Cat 1	Cat 2	Cat 3	Primary-Care Manageable (1 + 2), %
Acne/acne vulgaris	98	24.5	20	33	45	54
Dermatitis (atopic/contact/other)	73	18.2	35	13	25	66
Dermatophytosis/tinea/*Pityriasis versicolor*	39	9.8	23	8	8	79
Skin signs/symptoms (nonspecific)	35	8.8	4	6	25	29
Viral warts/molluscum	22	5.5	0	2	20	9
Seborrheic dermatitis	19	4.8	13	4	2	89
Alopecia/telogen effluvium	18	4.5	0	6	12	33
Pruritus	13	3.2	3	3	7	46
Scabies	11	2.8	5	1	5	55
Pigmentary disorders/vitiligo	10	2.5	0	2	8	20
Urticaria	10	2.5	1	3	6	40
Xerosis cutis	7	1.8	3	1	3	57
Psoriasis	5	1.2	0	1	4	20
Other skin/subcutaneous infections	5	1.2	0	3	2	60
Rosacea	4	1.0	0	4	0	100
Calluses and corns	4	1.0	0	0	4	0
Benign neoplasms (nevus/keratosis)	4	1.0	0	1	3	25
Nail disorders	3	0.8	0	1	2	33
Other	20	5.0	3	4	13	35
**Total**	**400**	**100**	**110**	**96**	**194**	**51.5**

Cat 1 = manageable by family physician; Cat 2 = manageable with teledermatology; Cat 3 = dermatology referral required. Percentages of the total may not sum to 100 because of rounding.

## Data Availability

The data supporting the findings are available upon reasonable request, subject to approval by the Istanbul Provincial Health Directorate.

## Supplementary Materials

The following supporting information can be downloaded at https://www.mdpi.com/article/10.3390/healthcare14111596/s1. Table S1. Diagnosis-level breakdown of all 400 presentations, with management-category distribution. Diagnoses recorded fewer than three times (and uncoded/compound entries) are aggregated into a single residual row so that the table reconciles exactly to the 400 cases and to the category totals of the main analysis (110/96/194). Cat 1 = family physician; Cat 2 = teledermatology-supported; Cat 3 = referral.
