# Supplementary material for: Identification of Dermatological Conditions Manageable in Primary Care: A Multidisciplinary Consensus Analysis
Source: Healthcare (Basel). 2026 Jun 5;14(11):1596. doi: 10.3390/healthcare14111596 (PMC13257235; doi:10.3390/healthcare14111596)
Supplement: Supplementary file 1 [file healthcare-14-01596-s001.zip › healthcare-4334161-supplementary.pdf]

Supplementary Material

Table S1. Diagnosis-level breakdown of all 400 presentations, with management-category distribution. Diagnoses recorded fewer than three times (and uncoded/compound entries) are aggregated into a single residual row so that the table reconciles exactly to the 400 cases and to the category totals of the main analysis (110 / 96 / 194). Cat 1 = family physician; Cat 2 = teledermatology-supported; Cat 3 = referral.

Diagnosis	n	Cat 1	Cat 2	Cat 3
Acne	62	15	22	25
Dermatitis, other	54	29	6	19
Acne vulgaris	30	3	8	19
Dermatophytosis	25	15	6	4
Viral warts	19	0	2	17
Skin changes, other	18	3	2	13
Seborrheic dermatitis	17	12	4	1
Nonspecific skin signs/symptoms	14	1	3	10
Pruritus	13	3	3	7
Scabies	11	5	1	5
Alopecia areata	10	0	0	10
Atopic dermatitis	8	5	3	0
Xerosis cutis	6	2	1	3
Telogen effluvium	5	0	3	2
Idiopathic urticaria	5	1	2	2
Melanin hyperpigmentation, other	4	0	1	3
Calluses and corns	4	0	0	4
Psoriasis	4	0	1	3
Rosacea	3	0	3	0
Urticaria	3	0	1	2
Pityriasis versicolor	3	3	0	0
Other / low-frequency diagnoses (each n < 3 or uncoded)	82	13	24	45
**Total**	**400**	**110**	**96**	**194**

Note: Groupings consolidate free-text ICD-10 descriptions; small overlapping/compound coded entries are aggregated into related categories or "Other / unclassified," so subgroup totals sum to 400.
